# A Note on Asthma
1A Paper read at a Meeting of the Bath and Bristol Branch of the British Medical Association, March 25th, 1925.


**Published:** 1925

**Authors:** J. R. Charles

**Affiliations:** Physician, Bristol Royal Infirmary


### A NOTE ON ASTHMA.[1]

BY

J. R. Charles, M.D., F.R.C.P.,

*Physician, Bristol Royal Infirmary.*

---

Of asthma, as is well known, there are very many etiological factors, *i.e.* underlying causes, many of which are hereditary, and also numerous exciting causes. From time to time special attention is devoted to one or other of these innumerable factors. At times the influence of metabolic disturbances holds the field ; at others the so-called exudative diathesis. More recently the attention of the physician has been devoted to sensitisation by all sorts of foreign proteins. Excess of various forms of food, *e.g.* acids and fats, various emotional disturbances, the influence of climate, and local affections of the nose, throat and so forth all play their part. All of these may produce attacks of spasmodic asthma.

Intrathoracic pressure may, however, be a determining factor, as *e.g.* in the case of a small aneurism, though as a rule the true cause of this will become manifest. The importance of seeking for some such cause has recently been stressed in a paper read before the Royal Society of Medicine by Lapage and Adams (November 24th, 1924). The point of some interest was the frequency with which they found intrathoracic glands and local lesions in the chest as determining factors, and they regard their action as not

[1] A Paper read at a Meeting of the Bath and Bristol Branch of the British Medical Association, March 25th, 1925.

entirely mechanical. Among 68 cases of asthma in children they found by clinical and X-ray examination definite lesions in 50, viz. :—

Bronchial glands in 31.
Mediastinal glands in 6.
Cervical glands in 5.
Fibrosis of root of lungs in 18.
Adhesions in 5.
Chronic bronchitis in 11.

One child suddenly coughed up large pieces of debris from a calcareous gland. Luckily he did not choke, and as a result of the mass coming away he was very much better and his asthmatic attacks subsided.

With regard to intrathoracic pressure, it might be supposed that the symptoms would be continuous, but a very considerable degree of pressure may be existent with prolonged freedom from any attacks, and the attacks when they do occur may be so typical of asthma produced by other causes that the true cause in the individual case may be readily overlooked.

In the case under observation the patient had always been a person with a very considerable degree of good breathing capacity, so that when at school he was able to do long distance cross-country runs with considerable ease, and sometimes beat records, and in the ordinary way, even with considerable exertion, had never had any shortness of breath. But throughout his life he had been subject at intervals to typical asthmatic attacks, by which I mean that, having gone to bed perfectly free and comfortable in breathing, he would wake up at 2 or 3 a.m. with severe dyspnœa and all the usual symptoms of asthma, which would clear up in the usual way, leaving him free from it the following day, perhaps to be repeated on subsequent nights.

On several occasions the dypsnœa had continued throughout the day and had been continuous, though worse, at night. Climate had a great deal to do with these attacks, *e.g.* on going to Cornwall some years ago the whole of this train of events occurred and remained persistent until he left. He went up to the Midlands, and immediately the whole condition subsided, and the conclusion was drawn that he was suffering solely from asthmatic trouble produced entirely by the climate. It was never suspected for one moment that he had any gross intrathoracic pressure. Sometimes it had been thought that exposure to the weather when motoring in an open car might have been an exciting cause of the attacks, but subsequent events threw great doubt on this supposition, because quite probably such attacks were produced by hemorrhage into an intrathoracic cyst following such exertions as winding up a refractory car, and not by exposure.

During last summer he noticed for the first time a slight enlargement of the thyroid which appeared just behind the right clavicle. Later on asthmatic attacks exactly the same as before became more frequent at night, and on one occasion the attack was so severe that he was unable to move at all for five hours, and then for the first time pressure was suspected as a possible cause. The attacks improved, but later on he had another, and was then X-rayed, which showed a definite shadow in the mediastinum on the right side bending the trachea over to the left, which proved to be due to an intrathoracic adenoma of the thyroid into which recent hemorrhage had taken place, which was successfully removed on November 26th, 1924, since which time no asthmatic attacks have occurred.

Both Mr. Berry, who did the operation, and Sir William

Hale White considered that this cyst was of very many years' duration and had been responsible for the whole of the trouble.

Here, then, was a case of typical asthmatic attacks occurring in the middle of the night, and definitely influenced by climate, which could only have acted as an accessory factor, the cause of which was entirely overlooked because it was never even suspected, but which could have been found almost certainly many years ago if the thought had occurred that the chest should be X-rayed; and therefore the example of this case is a plea for having an X-ray examination in any case of asthma which has not satisfactorily responded to treatment, to see if any form whatsoever of intrathoracic pressure may be at the root of the trouble.

Prolonged intermittency of attacks, nocturnal incidence of attacks, climatic influences on the attacks, and typical asthmatic characteristics of the attacks do not preclude the possibility of some such cause, as can quite definitely be learnt from this case.

The tumour which was removed by resection and enucleation was a well-encapsuled cystic adenoma weighing 6½ ozs. Microscopically it proved to be a simple unilocular colloid cyst, with recent hemorrhagic ooze into it. Many of the previous attacks of asthma had probably been due to similar hemorrhagic oozing following unusual exertion.

